# Evaluation of the quality of life and stress levels of parents/caregivers of adolescents and young adults with autism spectrum disorder in Serbia

**DOI:** 10.3389/fpsyt.2026.1750044

**Published:** 2026-02-03

**Authors:** Milica Vlaisavljevic, Vanja Mandic Maravic, Roberto Grujicic, Sanja Lestarevic, Jelena Vasic, Milica Pejovic-Milovancevic

**Affiliations:** Institute of Mental Health, Belgrade, Serbia

**Keywords:** autism spectrum disorder, families, quality of life, stress, support system

## Abstract

**Background:**

Families of individuals with autism spectrum disorder (ASD) may experience higher levels of stress and lower quality of life (QoL), taking into account their complex needs and daily challenges, especially when faced with discrimination and unavailable support services. This study aimed to examine the QoL and stress levels of parents/caregivers of adolescents and young adults aged 15–30 years and determine the relationship with support systems in Serbia.

**Subjects and methods:**

The study included 74 parents/caregivers of adolescents and young adults with ASD. The subjects were recruited from the database of the Institute for Mental Health in Belgrade. We used the following instruments: WHO Quality of Life-BREF, the Parental Stress Scale, and the Caregiver Needs Survey.

**Results:**

The obtained results show statistically significant relationships between stress levels and factors such as unavailable services (p=0.036), lack of information (p<0.0005), financial difficulties (p<0.0005), discrimination (p=0.004), and feelings of helplessness (p=0.018) due to the child’s ASD diagnosis. A negative correlation is found between all QoL domains and the level of stress, with p<0.0005 for physical health, psychological health, and environment domain and p = 0.001 for the domain of social relationships.

**Conclusion:**

The obtained data point out to the most significant socio-economic factors and factors within the support system in Serbia that affect the QoL and stress levels of parents/caregivers of youth with ASD. It can be used to create a more specific planning of organizational services, oriented towards the most problematic aspects of care for persons with ASD and their families.

## Introduction

Epidemiological studies and current estimates from the Centre for Disease Control and Prevention (CDC) indicate that 1 in 36 children is diagnosed with an autism spectrum disorder (ASD), a sharp increase over previous decades, potentially due to improved diagnosis and awareness of ASD (1, 2). These findings suggest that increasing public awareness of ASD can help ensure earlier diagnosis and timely intervention. It is also important to strengthen the support system for individuals with ASD and their families to ensure they receive appropriate help in managing the complex needs and challenges they encounter and ensure, as one of the goals, a better quality of life (QoL) and a lower level of daily stress. The World Health Organization (WHO) defines QoL as an individual’s perception of their general well-being, both mental and social, and not simply the absence of disease (3). The QoL of parents/caregivers of children with ASD can be significantly affected by the stress of everyday life. Parental stress is defined as stress caused by the perception of parents/caregivers that they do not have adequate competence to meet the demands arising from parenthood (4).

Studies show that parents/caregivers raising a child with a developmental disability (DD), including ASD, report lower QoL and higher levels of strain on the family system (5–7). Furthermore, Turnage and Conner found in an integrative review including 5,565 participants that higher parental education and milder ASD symptoms appear to be protective factors, although overall QoL remains reduced in this population (5). In addition, a systematic review and meta-analysis highlights important gaps in the existing literature regarding the QoL of parents of children with mental disorders, emphasizing the need for further research to better understand and support these families (6). Most recently research published in 2025 also indicates lower QoL and higher levels of stress among parents of children with ASD, although this study focused specifically on families of preschool-aged children, as most studies on this topic (8).

Parents/caregivers of individuals with ASD often doubt their parenting skills and poorly assess the functioning of the family as a whole. As the family is the main source of support for children with ASD, parents/caregivers must be empowered to provide care and face numerous and continuous challenges. One of potential solutions for improving the QoL of parents/caregivers is their increased involvement in interventions intended for children or adolescents with ASD, as well as targeted education programmes to improve knowledge of the disorder and gain skills for overcoming challenges (5, 9). A particularly important factor of the family QoL, apart from the severity of the disorder itself, is the availability of social support services for families (10). The wealth of a society is reflected in the ability to support its most vulnerable members. Research on parenting stress and QoL are often focused on the impact of disorder severity (5) or the characteristics of ASD, such as behavior problems or sensory difficulties (11–14), social and communication deficits/delays and problems in the parent-child relationship (15) and less on the influence of socio-cultural, economic factors and support systems within a country.

Moreover, most research is focused on families of young and school-age children with ASD, while research about adolescents/young adults with ASD and their families is relatively scarce (16), even though autism is a lifelong condition for family members and the affected person. Interventions for reducing stress in parents/caregivers and improving their QoL are a very important and often overlooked part of treating children with ASD. Therefore, addressing this research gap is crucial to expand knowledge on the needs, challenges and effective support strategies for families of adolescents and young adults with ASD.

In Serbia, new, improved guidelines for autism in children and adults were published in 2023, as an important tool for providing guidance and support in the daily work of professionals with families. One of the goals of these guidelines is to help families by improving the QoL of parents/caregivers (17). During 2022 and 2023, research was conducted in Serbia with the aim of identifying existing support systems intended for and available to families of adolescents/young adults with ASD, as well as their greatest needs and challenges. Results demonstrated that support for young people with ASD and their families declines during the transition to adolescence (18). Current research sought to investigate the effects of this decline on parents/caregivers’ stress and QoL. The aim of this study was to examine the QoL and stress level of parents/caregivers of adolescents and young people with ASD, aged 15 to 30 years. We also determined the relationship between the previously examined availability of existing support systems in Serbia and the challenges faced by families, with the QoL and stress levels of parents/caregivers. By identifying the most vulnerable aspect of the support system, we might decrease the level of stress and increase the QoL in parents/caregivers, as well as in families with children with ASD as a whole.

## Subjects and methods

### Study participants and procedure

This research is a part of a larger research that included the parents/caregivers of adolescents and young adults with ASD, examining support systems in Serbia (18). The present study focused on differences in stress levels and QoL according to variables related to accessibility and unmet needs over the past 12 months, the effect of ASD on the family, as well as the effect of stigmatization on the family. A total of 74 parents/caregivers of youth aged 15 to 30 years agreed to participate in this study. Inclusion criteria were being a caregiver of an individual aged 15–30 diagnosed with ASD diagnosis, voluntary participation in the study confirmed by the respondent signing an informed consent form, and the respondent’s ability to answer the questions. The exclusion criteria included participants’ refusal to take part in the study, their inability to complete the questionnaires due to health conditions that interfered with their ability to respond accurately, as well as cases where the child did not have an official ASD diagnosis or did not fall within the defined age range. Participants were recruited from the database of the Institute of Mental Health (IMH) in Belgrade and asked to participate during a regular visit. During regular follow-up visits, the treating physician informed eligible families about the study and referred those who expressed interest to the research team. The researchers then explained the purpose of the study, provided detailed information about participation and obtained written informed consent from all participants. Questionnaires were completed independently by parents/caregivers in the presence of a researcher, who was available to provide clarification or assistance if needed. This procedure minimized missing data and ensured standardized conditions for data collection. Recruitment of participants through the Institute of Mental Health enabled the identification of families with child with a confirmed ASD diagnosis, which was relevant to the aims of this study.

### Instruments

*The Serbian version of the WHO Quality of Life-BREF (WHOQOL-BREF)* (19, 20) was used to assess QoL among parents/caregivers. It is a self-assessment questionnaire consisting of 26 items. Each item is rated on a 5-point Likert scale, 1 meaning “not at all” and 5 “completely”. The higher the score, the better the QoL of the participant, while items number 3, 4 and 26 are scored in reverse. The items in the questionnaire can be grouped into four domains: physical domain (seven items), psychological domain (six items), social domain (three items) and environment domain (eight items). Two questions that are not classified into domains have a role in self-perception of QoL and general health. Domain scores are the sum of scores for each question within the domain, and finally, all scores are transformed into a range of 0–100 according to the WHO-BREF guidelines (21). In this study, we used only the domain scores. The total score was not calculated in accordance with the recommendations of the WHOQOL user manual, which does not recommend that the total QOL score be calculated by summing data from all WHOQOL items (20).

*The Parental Stress Scale* (22), measures the level of stress experienced by parents and takes into account both positive and negative “stressful” aspects of parenting. It contains various measures of stress, emotions, and satisfaction with the parental role. It is a self-report scale, containing 18 items rated on a 5-point scale (strongly disagree, disagree, undecided, agree, strongly agree). The higher the score, the higher the measured level of parental stress.

### Ethics

This research was approved by the Ethics Committee of the Institute of Mental Health in Belgrade (decision No. 1060/z027lt). All participants have signed the Informed Consent Form prior to inclusion in the research.

### Statistical analysis

Results for numerical variables are expressed as means ± SD, along with the number of observations (N), minimum and maximum values where applicable. For categorical independent variables, results are presented by category with corresponding N and mean ± SD of the dependent variable. The distribution of numerical variables and the suitability of the data for parametric tests were assessed prior to analysis using Shapiro-Wilk test. Comparisons between two groups were analyzed by ANOVA. Comparations between more than two groups were done using Bonferroni adjustment. The collinearity of the examined variables was verified using Pearson’s correlation test. Differences were considered significant at p < 0.05. Statistical analyses were conducted using the statistical software SPSS 17.0 (Statistical Package for the Social Sciences, Chicago, Illinois) for Windows.

## Results

The youth with ASD were mostly males (63.7%) with the average age of 20.05 ± 4.2. The average age of parents/caregivers was 51.07 ± 6.97. The majority of participants were mothers (82.4%) and the rest were fathers and one sister who was a caregiver. A little more than half of the parents/caregivers were married at the time of the interview (52.7%), while the other half were divorced, widowed, or a single caregiver. The majority of mothers (47.9%) and fathers (57.5%) had completed primary and secondary school, and 40% of mothers and 28.8% of fathers had university degree. Detailed socio-demographic characteristics of parents/caregivers and their children are reported and presented in a table in a previous publication (18).

**Table 1** shows the sum of scores for each domain in the WHOQOL-BREF (physical, psychological, social and environment domain) and the level of stress (The Parental Stress Scale).

**Table 1:** Descriptive statistics on QoL and stress level.

QoL domain	N	Minimum	Maximum	Mean	Std. Deviation
Physical domain	62	3.57	67.86	30.0115	15.32327
Psychological domain	65	33.33	91.67	61.3462	12.81097
Social domain	66	8.33	100.00	64.8990	20.75061
Environment domain	63	25.00	100.00	60.9623	14.33015
Level of stress	65	25.00	64.00	42.4615	10.17515

The results show no statistically significant differences between parental gender in terms of stress level, and each domain of QoL (data not shown).

Significant differences in stress levels were observed for participants reporting unavailable services, lack of information, feeling frustrated in the last 12 months in trying to get services in the support system in the country, financial difficulties, discrimination, and feeling helpless due to the child’s ASD diagnosis. The results are shown in **Table 2**.

**Table 2:** Relationship between level of stress and support systems.

Variable	Category	N	Level of stress (Mean ± SD)	ANOVA	p
Did not meet the criteria for a service	No	50	41.86 ± 10.15	0.753	0.475
	Yes	7	42.00 ± 11.21		
	I do not know	6	47.33 ± 11.27		
Unavailable services	No	53	41.32 ± 10.20	3.499	**0.036**
	Yes	7	51.71 ± 8.90		
	I do not know	5	41.60 ± 4.28		
Problems with waiting lists	No	48	41.89 ± 9.28	0.958	0.389
	Yes	14	43.71 ± 12.99		
	I do not know	2	51.50 ± 10.61		
Problems with cost of services	No	57	42.21 ± 10.24	0.173	0.842
	Yes	3	43.00 ± 13.75		
	I do not know	5	45.00 ± 9.27		
Lack of information	No	42	38.88 ± 9.24	10.898	**< 0.0005**
	Yes	19	50.00 ± 8.45		
	I do not know	2	50.50 ± 4.95		
Frustration in efforts to obtain services	Never,sometiomes	48	39.77 ± 9.15	7.790	**0.001**
	Usually, always	15	49.93 ± 9.81		
	Don’t know	2	51.00 ± 4.24		
Financial difficulties	No	31	38.58 ± 8.95	9.124	**< 0.0005**
	Yes	32	47.06 ± 9.36		
	I do not know	2	29.00 ± 1.41		
Quittinq work	No	29	39.89± 9.82	1.810	0.172
	Yes	35	44.66 ± 10.23		
	I do not know	1	40.00		
Feeling helpless	I do not agree at all	11	36.73 ± 6.53	3.610	**0.018**
	I do not agree	26	40.88 ± 10.22		
	I agree	18	44.22 ± 10.23		
	I totally agree	10	49.70 ± 9.44		
Concerns about autism	I do not agree at all	47	41.36 ± 10.29	1.603	0.210
	I do not agree	17	44.71± 9.45		
	I agree	1	56.0		
Discrimination	I do not agree at all	16	37.75 ± 8.15	4.952	**0.004**
	I do not agree	32	41.12 ± 9.39		
	I agree	12	50.67 ± 8.74		
	I totally agree	5	46.40 ± 13.76		
Negative influence	I do not agree at all	18	33.61 ± 5.36	11.952	**< 0.0005**
	I do not agree	22	42.36 ± 7.09		
	I agree	19	48.95 ± 10.00		
	I totally agree	6	48.83 ± 12.89		

Bolded values are statistically significant.

**Table 3** presents variables from **Table 2** for which significant differences in QoL were observed. These include financial difficulties, discrimination, lack of information, as well as “feeling helpless because the child has a diagnosis of ASD”.

**Table 3:** Relationship between QoL domains and support systems.

Variable	Category	N	Mean ± SD	ANOVA	p
Physical health (QoL)					
Financial difficulties	No	31	113.29 ± 15.26	5.886	**0.005**
	Yes	33	99.76 ± 16.49		
	I do not know	2	104.00 ± 5.66		
Discrimination	I do not agree at all	15	116.53 ± 17.62	5.242	**0.003**
	I do not agree	33	107.39 ± 13.31		
	I agree	13	92.92 ± 17.60		
	I totally agree	5	92.00 ± 16.97		
Feeling helpless	I do not agree at all	11	113.82 ± 13.78	3.405	**0.023**
	I do not agree	26	107.08 ± 17.65		
	I agree	18	108.44 ± 14.64		
	I totally agree	11	93.09 ± 16.40		
Negative influence	I do not agree at all	18	113.33 ± 14.71	3.582	**0.019**
	I do not agree	23	109.22 ± 16.33		
	I agree	19	99.58 ± 18.37		
	I totally agree	6	94.67 ± 7.45		
Psychological health (QoL)					
Financial difficulties	No	32	92.25 ± 13.63	4.156	**0.020**
	Yes	31	81.94 ± 17.40		
	I do not know	2	100.00 ± 5.66		
Discrimination	I do not agree at all	15	96.80 ± 14.36	3.631	**0.018**
	I do not agree	33	87.39 ± 12.09		
	I agree	12	77.33 ± 22.65		
	I totally agree	5	85.60 ± 16.64		
Negative influence	I do not agree at all	18	97.56 ± 10-55	11.934	**<0.0005**
	I do not agree	23	92.17 ± 10.12		
	I agree	18	72.89 ± 17.76		
	I totally agree	6	84.00 ± 14.97		
Social relations (QoL)					
Lack of information	No	44	45.36 ± 9.00	3.540	**0.035**
	Yes	19	38.36 ± 11.26		
	I do not know	1	40.00		
Financial difficulties	No	32	45.87 ± 7.74	4.068	**0.022**
	Yes	32	39.87 ± 11.11		
	I do not know	2	52.00 ± 5.66		
Negative influence	I do not agree at all	18	47.11 ± 7.33	3.077	**0.034**
	I do not agree	23	44.17 ± 7.48		
	I agree	19	37.89 ± 13.29		
	I totally agree	6	44.00 ± 7.59		
Environment (QoL)					
Lack of information	No	42	114.48 ± 18.64	5.744	**0.005**
	Yes	19	99.16 ± 13.37		
	I do not know	1	128		
Financial difficulties	No	31	166.77 ± 18.49	4.68	**0.022**
	Yes	30	102.93 ± 16.24		
	I do not know	2	112.00 ± 6.66		
Feeling helpless	I do not agree at all	10	122.49 ± 15.57	3.641	**0.018**
	I do not agree	24	112.40 ± 15.15		
	I agree	18	107.78 ± 18.68		
	I totally agree	11	98.18 ± 20/50		

The bolded values indicate the values to be statistically significant.

Statistically significant results of the examined correlation between stress level and QoL are shown in **Table 4**. A negative correlation was found across all QoL domains.

**Table 4:** Correlations between stress level and QoL.

Variable	N	Level of stress
Physical health	60	r = -0.479, **p < 0.0005**
Psychological health	64	r = - 0.628, **p < 0.0005**
Social relations	64	r = - 0.407, **p = 0.001**
Environment	61	r = - 0.443, **p < 0.0005**

The bolded values indicate the values to be statistically significant.

## Discussion

The study aimed to evaluate the QoL and stress levels of parents/caregivers of adolescents and young adults with ASD and how national support systems affect these outcomes. This study is particularly important and original as it focuses on adolescents and young adults with ASD in Serbia, a population that has been largely underrepresented in previous research, thereby addressing a critical research gap and providing valuable insights into parental QoL and stress in this context.

Demographic findings were consistent with previous researches, showing that mothers were typically the primary caregivers involved in childcare and contacts with professional staff, and that males predominated among youth with ASD (23, 24). Nearly half of the participants were divorced, widowed, or single, supporting earlier findings that parents/caregivers of children with ASD have higher divorce rates (25). Research on support shows that parents perceive partners and family as the greatest support, greater than professionals, but family support appears to decline with the age of the parent and child (26). In order to preserve the family integrity it is important to implement family-oriented support and partner-focused interventions.

The lowest QoL scores were found in the physical domain, likely reflecting parental/caregiver exhaustion and reduced self-care due to continuous caregiving demands with consequent chronic fatigue, lower every day and work efficiency as well as sleep disorders. Compared to parents/caregivers of children with typical development, parents/caregivers of individuals with ASD have higher fatigue, poor sleep quality and poor quality of physical activity which all affect their wellbeing (27). Parents/caregivers in this study reported lower scores across all QoL domains, although no normative values are currently available for the general population in Serbia. The average stress level indicates moderately high stress, consistent with researches showing higher stress among parents/caregivers of children with ASD compared to parents/caregivers of children without developmental disabilities (7, 28, 29). Recent researches involving parents of young children with ASD suggest that high parental stress is evident in early parenthood and is associated with reduced QoL across multiple domains, highlighting that stress and QoL challenges emerge early (8, 30). When considered in the context of findings from this study, these challenges may persist continuously through adolescence and young adulthood. International studies from other countries, like Australia, Norway and Denmark have also examined QoL and stress in this population, providing contextual background for understanding these findings (31–33), similar to the contribution of the present study within the Serbian context. Although there were no significant differences between mothers and fathers in the results of QoL assessments, different dimensions of family functioning and gender roles that were not analyzed in this study could be further explored to better understand the specific differences in parenting experiences and their impact on QoL.

Statistically significant differences were found for reported variables such as financial difficulties, discrimination, feelings of helplessness, and perceived negative impact of ASD on the family, with higher levels of stress and lower scores across various domains of QoL. Many parents/caregivers had to quit or reduce work hours due to their child’s needs (18), which is consistent with earlier studies on employment and income challenges in ASD families (34–36). Additionally, 68% of adolescents and young adults with ASD did not attend daily care centers (17), forcing parents/caregivers to balance caregiving and employment with little time for rest or social life. The high cost of specific care treatments also represents a financial strain. These findings align with the National Autism Indicators Report, which shows increased material hardship in ASD families, including those with adolescents (37). Discriminaton is a very significant topic and problem in society and the obtained data indicate the importance of systemic changes in order to promote the inclusion of people with ASD and their families while raising awareness in society about ASD, in order to work on preventing stigmatization and discrimination of this vulnerable population group and thus improve their well-being and reduce the stress related to isolation.

The results also indicate the role of psychosocial well-being in physical health, as helplessness may manifest in fatigue, reduced functioning, and sleep problems. Daily challenges of caring for individuals with ASD can lead to chronic exhaustion, reduced subjective well-being and limited opportunities to participate in social activities, along with experiencing reduced interactions and support. All of this may contribute to their increased feelings of isolation. However, the 2023 scoping review found no targeted interventions for parents/caregivers of adults with ASD, indicating a clear support gap (38). Within the context of healthcare, social and educational systems, a lack of available information, unavailability of services and parental frustration related to attempts to access services over the past 12 months show statistically significant differences in stress levels and QoL. Lack of information was highlighted as one of the common reasons for the unavailability of services (18). The data confirm that inadequate information provided by professionals significantly affects parental/caregiver wellbeing and that service deficiencies and organizational barriers significantly limit access to necessary information (39–42). Strong communication and collaboration between parents and teachers can provide families with key information, security and access to relevant resources. Hsiao and colleagues (40) emphasize that positive interactions with teachers and school staff can help alleviate parents’ feelings of helplessness and stress by providing a more structured and supportive environment for their child. In addition to improving the availability of services, strengthening cooperation between professionals and parents in the educational environment can play a key role in improving their well-being and consequently QoL. The data obtained are very important from the aspect of organizing the work of the national support system, given the significance of accessibility of relevant information and professional help for reducing their stress (43). Informational resources as part of the environment in which the family lives can be an effective tool in helping parents/caregivers cope with the challenges and stress commonly experienced in the context of caring for a person with ASD. The findings further emphasize the importance of social roles, aligning with previous studies that highlight the critical role of social support in mitigating parental stress and indicate that access to confidant and emotional support is particularly beneficial for parents of children with ASD (44, 45).

The results of the correlation analysis indicate statistically significant negative correlations between stress levels and all domains of QoL, with the strongest negative correlation observed between stress levels and psychological health. These findings further support the argument that stress is a critical factor affecting overall QoL, not only at the individual level but also within the broader family context. Most studies about the degree and factors of burden on families of adults with ASD have shown that parents/caregivers primarily highlighted the emotional burden of caring for an adult with ASD, along with reduced psychological QoL (38). Also, an increased level of stress tends to coincide with a reduced ability of parents/caregivers to maintain interpersonal relationships, which negatively affects perceptions of the environment in which they live, like the availability of resources, safety and support. If parents/caregivers are involved in psychological support groups, have a stronger social network, connections with other parents or manage to integrate social activities with family needs, this can significantly improve their QoL and reduce stress levels. Social support is one of the key factor related to parental stress and QoL and can serve as a resource for implementing interventions and designing support strategies (46, 47). In the domain of physical health, chronic stress can have negative effects which can be explained by the allostatic load theory, that describes how prolonged activation of physiological stress responses leads to cumulative damage to the body (48). Overall, the findings highlight the need for holistic interventions that enhance parental mental health and provide continuous, accessible support for families across the lifespan of individuals with ASD. Such interventions should focus on improving access to information, strengthening collaboration between families and professionals within healthcare, education and social services and raising public awareness of the challenges faced by this population, ultimately promoting the psychological well-being of parents/caregivers and reducing stress.

## Limitations

This study has several limitations. One limitation is the small, convenient sample of parents/caregivers from urban areas, most of whom are already involved in various programs and support services which may reduce the generalizability of the results. Additionally, those who were willing to participate likely feel less burdened by stigma. The majority of participants were mothers, who are typically more involved in caring for children with ASD in Serbia, further limiting the generalizability of the findings. A limitation is also the absence of normative data for QoL scores in the general population in Serbia, which prevents direct comparisons. The study does not compare parents/caregivers of children with ASD to those of typically developing children, which would provide a clearer understanding of the specific challenges faced by families of children with ASD.

## Conclusion

The QoL and stress levels experienced by families of youth with ASD is a current topic that is attracting the attention of experts worldwide. Research in this area should provide better insight into the specific needs and challenges in order to create more effective support strategies and improve available systems and resources. This study aimed to identify specific domains of QoL and stress levels among parents/caregivers of adolescents and young adults with ASD as a potential basis for planning interventions that can increase QoL and improve family resilience. Given the epidemiological data indicating a significant increase in the population diagnosed with ASD, and therefore an increase in the number of parents/caregivers of people with ASD, research that will contribute to their better well-being and support is one of the priority issues in this field due to their lower QoL and higher level of stress. The contribution of this study lies in providing the first results addressing quality of life and stress levels among families of adolescents and young adults with ASD in Serbia. Understanding these patterns in this population is important not only locally, but also contributes to a better global understanding of caregiver and parent experiences, particularly given the limited number of studies on adolescents and adults with ASD and their families. These findings can also directly inform the development of targeted support strategies and interventions in Serbia. Given that the sample includes primarily service users living in urban areas, the question arises as to what the levels of QoL and stress are among families living in rural parts of the country, where available support systems and the number of professionals are more limited. Accordingly, a recommendation for future research is to further investigate the QoL and stress levels among families with youth with ASD in rural parts of the country. There is also a need for research about interventions that would contribute to greater well-being for this population group. This research highlights important socio-economic factors and significant factors within the support and protection system in the country, that can serve as a basis for further planning of the work of organizational services and public health policy.

## Data Availability

The raw data supporting the conclusions of this article will be made available by the authors, without undue reservation.
